# VEGFR2 Promotes Metastasis and PD-L2 Expression of Human Osteosarcoma Cells by Activating the STAT3 and RhoA-ROCK-LIMK2 Pathways

**DOI:** 10.3389/fonc.2020.543562

**Published:** 2020-09-09

**Authors:** Bingxin Zheng, Chuanli Zhou, Guojian Qu, Chongmin Ren, Peng Yan, Wei Guo, Bin Yue

**Affiliations:** ^1^Department of Orthopedic Oncology, The Affiliated Hospital of Qingdao University, Qingdao, China; ^2^Department of Spinal Surgery, The Affiliated Hospital of Qingdao University, Qingdao, China; ^3^Department of General Surgery(adult), Qingdao Women and Children’s Hospital, Qingdao, China; ^4^Musculoskeletal Tumor Center, Peking University People’s Hospital, Beijing, China

**Keywords:** VEGFR2, PD-L2, STAT3, metastasis, osteosarcoma

## Abstract

The survival rate of osteosarcoma, the most prevalent primary bone tumor, has not been effectively improved in the last 30 years. Hence, new treatments and drugs are urgently needed. Antiangiogenic therapy and immunotherapy have good antitumor effects in many kinds of tumors. It is hypothesized that there may be a synergistic effect between immune checkpoint inhibitors and antiangiogenic therapy. Nevertheless, its potential mechanism is still unclear. Vascular endothelial growth factor receptor-2 (VEGFR2) expression was detected by immunohistochemistry in 18 paired osteosarcoma tissues. Moreover, we investigated the effects of apatinib treatment and VEGFR2 knockdown on osteosarcoma as well as the relevant underlying mechanism. Immunohistochemistry assays showed that, compared with that in primary osteosarcoma, VEGFR2 expression was higher in lung metastases. VEGFR2 was positively correlated with PD-L2 expression in osteosarcoma lung metastasis. Transwell assays indicated that VEGFR2 inhibition reduced osteosarcoma cell metastatic abilities *in vitro*. We also demonstrated that VEGFR2 inhibition downregulated the STAT3 and RhoA-ROCK-LIMK2 pathways, thereby attenuating migration and invasion. Additionally, VEGFR2 inhibition targeted STAT3, through which it reduced PD-L2 expression in osteosarcoma cells. VEGFR2 inhibition markedly attenuated osteosarcoma lung metastatic ability *in vivo*. In this study, we presented the pro-metastatic functional mechanism of VEGFR2 in osteosarcoma. VEGFR2 inhibition exhibits antitumor effects through antiangiogenic effects and inhibition of immune escape, which possibly provides potential clinical treatment for metastatic osteosarcoma.

## Introduction

As a primary malignant bone tumor that is commonly seen in children and teenagers, osteosarcoma exhibits an obvious tendency toward locally aggressive and pulmonary metastasis (1–3). Doctors mainly rely on integrating neoadjuvant chemotherapy with surgery to treat osteosarcoma. Nevertheless, chemotherapy for osteosarcoma reaches a plateau period, and the 5-year survival of pulmonary metastases patients is only 28% (4). Thus, it is urgent to develop novel and effective treatments for osteosarcoma, particularly for patients with metastatic osteosarcoma.

Both programmed death ligand-1 (referred to as PD-L1) and programmed death ligand-2 (referred to as PD-L2) are the ligands of programmed death-1 (PD-1), and their combination leads to T-cell exhaustion and immune escape. Recently, immune checkpoint inhibitors (ICIs) have achieved good curative effects in varying solid tumors. However, most studies mainly center on the role of PD-1 ligands in the reciprocity between T-cells and tumor cells, and few studies have been performed on the intrinsic effects of PD-1 ligands in tumor cells, which may be associated with ICI treatment effects (5, 6). According to some studies, PD-L1/PD-L2 are associated with cellular biological behaviors such as autophagy, metastasis, propagation, and epithelial-mesenchymal transition (EMT) (7–11).

More recently, VEGFR2 inhibition with apatinib has shown remarkable effects in the treatment of multiple tumors (12–15). Moreover, some researchers have hypothesized that the integration of antiangiogenic therapy with ICI immunotherapies is likely to produce a collaborative result (16). However, the deeper and specific underlying mechanism of VEGFR2 affecting the metastasis and tumor immunity of osteosarcoma remain poorly understood.

We mainly addressed three aspects in this study: (1) VEGFR2 expression in primary and metastatic osteosarcoma; (2) significance of VEGFR2 inhibition to migration and invasion of osteosarcoma cells both *in vivo* and *in vitro*; and (3) possible mechanism of osteosarcoma metastasis mediated by VEGFR2.

## Materials and Methods

### Osteosarcoma Cell Samples and Patient Data

Primary osteosarcoma specimens fixed with formalin and embedded in paraffin and paired pulmonary metastasis specimens were acquired from the Musculoskeletal Tumor Center at Peking University People’s Hospital (Beijing, China) with relevant informed patient consent. To carry out this study, we also obtained approval from the Ethics Committees of The Affiliated Hospital of Qingdao University and Peking University People’s Hospital.

### Cell Growth and Reagents

KHOS and U2OS cells were obtained from American Type Culture Collection (ATCC), and we used short tandem repeat (STR) analysis for the authentication of the two cells at Beijing Microread Genetics Co., Ltd. Cells were cultured in RPMI 1640 medium (HyClone) supplemented with 1% penicillin and streptomycin (Invitrogen) and 10% fetal bovine serum (Gibco) in an environment with a temperature of 37°C and 5% CO2.

The antibodies we used in relevant experiments were as follows: antibodies against p-STAT3, STAT3, p-cofilin, cofilin and VEGFR2, which we purchased from Cell Signaling Technology in United States; anti-PDL2, p-LIMK2, LIMK2 and the RhoA activation assay, which we obtained from Abcam in United States; and anti-GAPDH, which we purchased from Santa Cruz Biotechnology. Y-27632 2HCl was purchased from Selleck.

### Gene Knockdown With siRNA/shRNA and Ectopic Expression

The lentiviruses targeting VEGFR2 and control shNC were obtained from GenePharma (Suzhou, China), and two pairs of shVEGFR2 primers were designed: #1, sense strand 5′-CTCG GTCATTTATGTCTAT-3′ and antisense strand 5′-ATAGACA TAAATGACCGAG-3′ and #2, sense strand 5′-CTTCGAAGCA TCAGCATAA-3′ and antisense strand 5′-TTATGCTGATG CTTCGAAG-3′. After infection with lentivirus, puromycin (2 μg/ml) was used to select cells stably expressing shVEGFR2 and shNC.

The source of siSTAT3 and control siRNA was GenePharma (Suzhou, China), and the detailed sequences targeting STAT3 were as follows: #1, 5′-AAAGAAUCACAUGCCACU UTT-3′ (sense) and 5′-AAGUGGCAUGUGAUUCUUUGC-3′ (antisense); #2, 5′-ACAAUCUACGAAGAAUCAATT-3′ (sense) and 5′-UUGAUUCUUCGUAGAUUGUGC-3′ (antisense); and #3, 5′-CGUCCAGUUCACUACUAAATT-3′ (sense) and 5′-UUUAGUAGUGAACUGGACGCC-3′ (antisense). SiRNAs were transfected into tumor cells by using Lipofectamine 3000 obtained from Invitrogen, United States, as per the protocols of the manufacturer. Further analyses were carried out on the cells collected after 48 h of transfection.

The plasmid containing VEGFR2 cDNA or negative control was transfected into KHOS cells with Lipofectamine 3000 (Invitrogen). The medium was replaced after 24 h incubation, and then the cells were treated with Y-27632 2HCl.

### Cell Counting Kit 8 Assay

After 2000 cells were seeded in each well of 96-well plates, the cells were treated with apatinib at various concentrations. The viability analysis was carried with CCK-8 (Dojindo Laboratories, Kumamoto, Japan) reagent as per the protocols of the manufacturer.

### Cell Colony Formation Assay

Cells (1 × 10^3^) in the logarithmic growth phase were coated into six-well plates. After forming colonies for 10 days, cells were fixed with methanol and stained with gentian violet staining solution.

### Transwell Assay

A total of 6 × 10^4^ cells were plated into the upper part of the Transwell insert (8 μm pore size; Corning) in the absence of coating for the migration assay or in the presence of a Matrigel coat (BD Bioscience, 354234) for the invasion assay. After 24 h of culture, the cells were fixed with methanol and stained with 0.1% gentian violet staining solution. The quantities of migrated cells were calculated with a microscope in 5 fields per well.

### Quantitative RT-PCR

Total RNA was obtained from KHOS and U2OS cells with TRIzol (Invitrogen). Then, complementary DNA (cDNA) was generated with isolated RNA and oligo dT primers by using SuperScript III First-Strand Synthesis SuperMix (Invitrogen). Real-time quantitative PCR was conducted with the SYBR Green PCR Master Mix from Applied Biosystems, United States and was run on Bio-Rad CFX96 following the manufacturer’s instructions.

### Bioinformatics Assay

The osteosarcoma dataset from GEO was used for the data mining and bioinformatics studies (Nos. GSE21257 and GSE33382). Cluster analysis and heat map generation were performed by MeV software (version 4.9). The association of the genes of interest and confirmed gene sets was demonstrated by using gene set enrichment analysis (GSEA). REVIGO was used for the gene annotation network analysis.

### Western Blotting and GTPase Assay

Western blotting was conducted as described previously (12). Briefly, the same volume of protein obtained from different cell lysates was loaded onto 7.5–15% SDS-PAGE gels and then transferred onto PVDF membranes. The membranes were cultured with suitable antibodies overnight at 4°C after blocking in non-fat milk for 1 h. The western blot bands were observed with a Bio-Rad detection system (Hercules, CA, United States). The GTPase assay was performed as per the manufacturer’s instructions.

### Immunohistochemistry Assay

Immunohistochemistry (IHC) staining was performed as previously described (12). Briefly, paraffin slices were incubated with corresponding primary antibodies or with non-immune serum in PBS overnight at 4°C. Then, the sections were stained with secondary antibody (ZSGB-BIO, China) for 30 min at 37°C. The percentage of positive cells was defined as follows: 0: 0% positive; 1: less than 5% positive; 2: between 5 and 50% positive; and 3: more than 50% positive. In addition, the staining intensity was scored as 4 follows: 0: no staining; 1: poor; 2: medium; and 3: intense. The total score was calculated by multiplying the intensity result by the percentage result. More than 10 representative fields (x400 magnification) were scored between the primary osteosarcoma and lung metastasis specimens for IHC analyses. The immunostaining was scored by two pathologists who were blinded to the clinical features of the clinical specimens.

### Immunofluorescence Assay

Cells (2 × 10^5^) were plated onto coverslips in six-well plates with or without the corresponding inhibitor treatment. The coverslips were cultured with 100 nM rhodamine phalloidin (manufactured in the United States) at room temperature for 45 min. The sections were observed by a confocal microscope manufactured in Japan.

### Tumor Xenografts

To confirm the effect of apatinib treatment on the metastatic ability of KHOS cells, 3 × 10^6^ KHOS cells were injected intravenously into the tail vein of 42-day-old maternal BALB/c nude mice from Vitalriver, China. On the 5th day after injection, the mice were randomly separated into two group (*n* = 5 per group)and fed DMSO or apatinib 2 mg/kg/day for 1 month total.

To evaluate the effect of VEGFR2 knockdown on the metastatic ability of KHOS cells, 42-day-old maternal BALB/c nude mice were intravenously injected with 3 × 10^6^ KHOS-shVEGFR2 or -shNC cells (*n* = 5 per group) via the tail vein. After 30 days, all mice were sacrificed.

At the end of the experiment, the lungs were collected, imaged, stained with haematoxylin-eosin dye, and the quantity of pulmonary metastases was confirmed. In addition, animal care and processing procedures were conducted in accordance with the National Institutes of Health Guide for the Care and Use of Laboratory Animals.

### Statistical Analysis

The statistical analysis was conducted by SPSS software (version 21.0). Statistical evaluation was conducted by Student’s *t*-test. Data are shown as the mean ± S.D. A *p*-value below 0.05 showed that there were significant differences.

## Results

### VEGFR2 Expression Is Elevated in Osteosarcoma Lung Metastases

**FIGURE 1:**
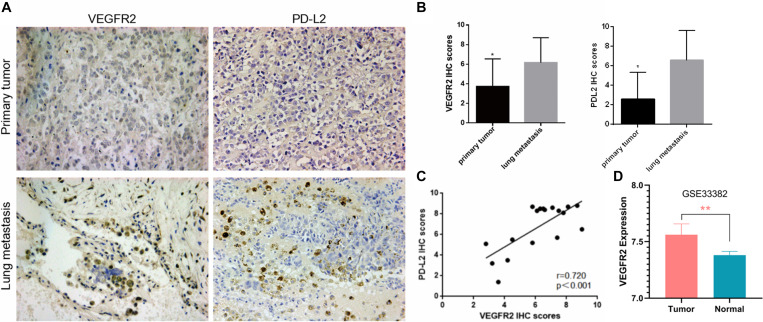
Elevated VEGFR2 expression in osteosarcoma lung metastasis. **(A)** VEGFR2 and PD-L2 expression in 18 pairs of primary osteosarcoma specimens and pulmonary metastasis specimens was investigated via immunohistochemistry. Representative images are presented (magnification at 200×). **(B)** IHC scores of VEGFR2 and PD-L2 were analyzed between the primary osteosarcoma group and the pulmonary metastasis group. **(C)** The correlation between PD-L2 and VEGFR2 expression levels in 18 osteosarcoma pulmonary metastases was investigated. **(D)** VEGFR2 was overexpressed in osteosarcoma in comparison to normal control. Data are shown as the mean ± S.D. *p < 0.05, **P < 0.01.

An IHC assay for VEGFR2 was conducted on paired primary osteosarcoma specimens and pulmonary transfer specimens. VEGFR2 exhibited cytoplasmic and nuclear expression (Figure 1A), and VEGFR2 expression was increased in pulmonary metastases compared with local osteosarcoma samples (Figure 1B), implying that VEGFR2 may play a significant role in osteosarcoma metastasis.

PD-L2 expression was also investigated in these paired osteosarcoma specimens, and the results indicated that PD-L2 expression was also elevated in lung metastasis compared with primary osteosarcoma (Figure 1A). Thus, we investigated whether VEGFR2 and PD-L2 were related to each other in osteosarcoma lung metastasis, and an important positive relationship between VEGFR2 and PD-L2 expression was confirmed (Figure 1C). VEGFR2 was overexpressed in osteosarcoma in comparison to normal control (Figure 1D).

Hence, the results reveal that VEGFR2 may play a crucial role in metastasis and immune escape of osteosarcoma.

### VEGFR2 Inhibition Suppresses the Migration and Invasion of Osteosarcoma Cells *in vitro*

**FIGURE 2:**
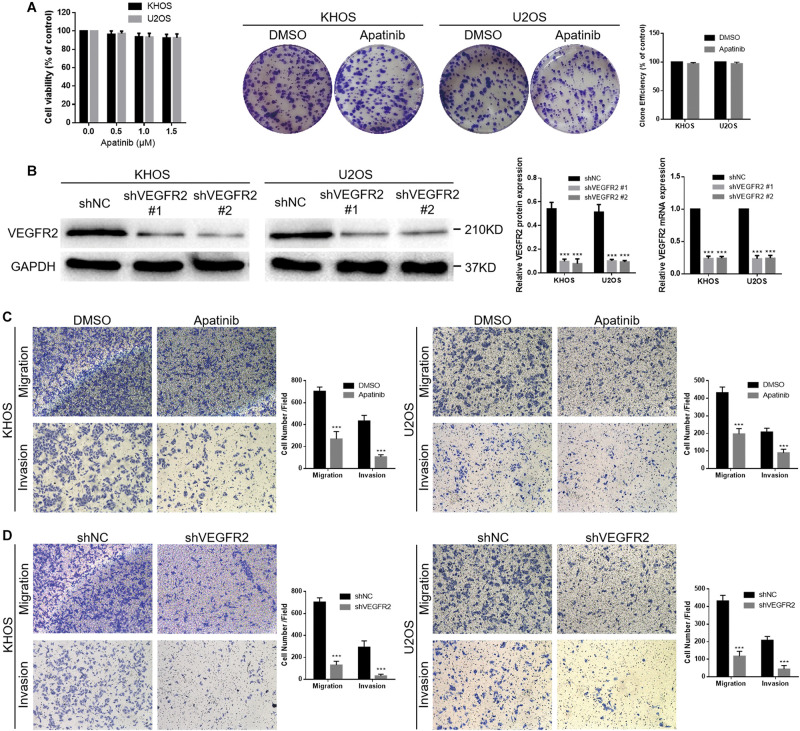
VEGFR2 inhibition decreases osteosarcoma cell invasion and migration. **(A)** Apatinib did not obviously influence KHOS and U2OS cell viability at concentrations of 0.5, 1.0, and 1.5 μM. Proliferation of these two cell lines in response to apatinib treatment was confirmed via cell colony formation analysis. **(B)** Both shNC and shVEGFR2 lentiviruses were transferred into KHOS and U2OS cells, following estimation of VEGFR2 expression via WB and RT-PCR. **(C,D)** Both apatinib treatment and VEGFR2 knockdown significantly attenuated the KHOS and U2OS cell migratory and invasive abilities, as demonstrated by a Transwell assay. (Magnification at 100×), (mean ± S.D., *n* = 3), ***p < 0.001.

The proliferation activity of KHOS and U2OS cells was not appreciably influenced by apatinib at concentrations of 0.5, 1.0, and 1.5 μM using the CCK-8 assay (Figure 2A). The 1.5 μM concentration was chosen as the upper limit for subsequent studies. There were few differences between the DMSO and apatinib groups in the cell colony formation analysis (Figure 2A). The two cell lines were transferred with lentivirus targeting VEGFR2, and VEGFR2 expression was later investigated using WB and real-time PCR assays (Figure 2B). Furthermore, we investigated the effect of VEGFR2 reduction on the migration and invasion of osteosarcoma cells using a Transwell assay.

The results showed that apatinib reduced the abilities of KHOS and U2OS cells to migrate and invade (Figure 2C). Moreover, both the KHOS-shVEGFR2 and U2OS-shVEGFR2 groups showed lower migratory and invasive abilities than the control groups (Figure 2D).

In short, these results indicate that VEGFR2 is crucial for the migratory and invasive abilities of osteosarcoma cells, and its specific molecular mechanism needs further study.

### Potential Mechanisms That Underlie VEGFR2 Associations With Metastasis

**FIGURE 3:**
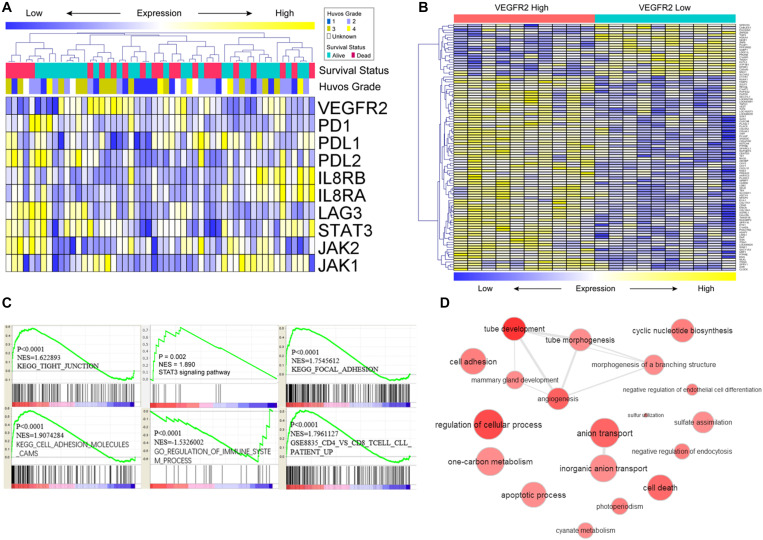
Potential mechanisms that underlie the VEGFR2 associations with metastasis and survival time. **(A)** VEGFR2-related genes, HUVOS grade and overall survival were analyzed by heat map observation and cluster analysis. **(B)** Differentially expressed genes were analyzed by visual analysis of heat maps in the VEGFR2 high- and low-expression groups. **(C,D)** Differentially expressed VEGFR2-related genes were investigated by analysis of the gene annotation network and GSEA.

A review of the expression of VEGFR2-related genes and their relationships with overall survival and HUVOS grade in osteosarcoma specimens from the GEO dataset is shown in Figure 3A. To investigate the potential mechanisms of the relationship of VEGFR2 with metastasis and prognosis, functional enrichment and gene expression of VEGFR2-related genes were calculated and visualized. The heat map of the top differentially expressed genes between the VEGFR2 high-expression group and the VEGFR2 low-expression group is shown in Figure 3B.

The GSEA results indicated that VEGFR2 was positively associated with STAT3 signaling pathway, activation of immune response pathways, cytoskeletal arrangement and relevant metastasis characteristics (Figure 3C). Gene annotation network analysis was conducted on the differentially expressed genes (Figure 3D: VEGFR2 high against VEGFR2 low).

These results show that VEGFR2 is related to immunosuppression, cytoskeletal arrangement and relevant metastasis pathways. Next, we investigated the specific mechanism by which VEGFR2 affects metastasis and immune escape *in vitro*.

### VEGFR2 Inhibition Inactivates RhoA-ROCK-LIMK2 Signaling and Suppresses PD-L2 Expression in Osteosarcoma Cells by Targeting STAT3

**FIGURE 4:**
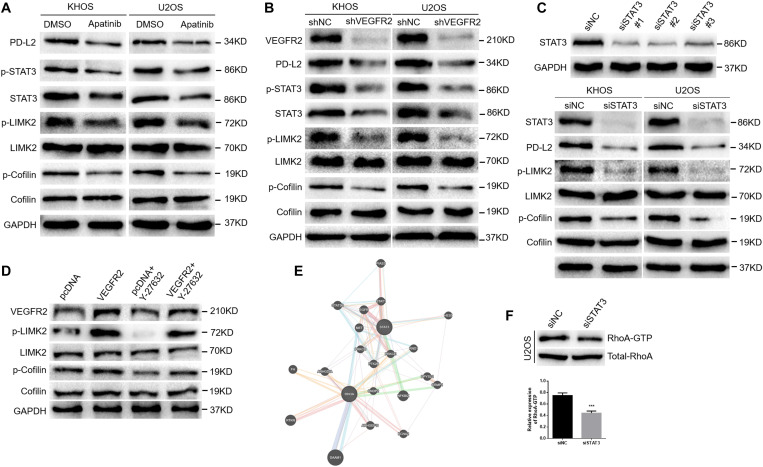
VEGFR2 inhibition inactivates the RhoA-ROCK-LIMK2 pathway and suppresses PD-L2 expression in osteosarcoma cells by targeting STAT3. **(A)** Apatinib reduced the expression of PD-L2, STAT3, p-cofilin and p-LIMK, as demonstrated by western blotting. **(B)** VEGFR2 knockdown reduced the expression of PD-L2, STAT3, p-cofilin and p-LIMK, as indicated by western blotting. **(C)** STAT3 expression in KHOS cells was downregulated by three STAT3 siRNA sequences (top). STAT3 knockdown decreased the expression of PD-L2, p-LIMK and p-cofilin (bottom). **(D)** Overexpression of VEGFR2 increased Y-27632 2HCl-induced decline of p-LIMK and p-Cofilin by western blot. **(E)** A coexistence phenomenon was observed between RhoA and STAT3 by bioinformatics analysis (http://genemania.org/). **(F)** The change in RhoA activation in U2OS cells was confirmed by the GTPase test. ***P < 0.001.

As Figure 4A shows, apatinib treatment reduced PD-L2 expression in KHOS and U2OS cells. Combined with the above IHC results, these findings showed that VEGFR2 significantly affected PD-L2 expression in osteosarcoma. A previous study suggested that PD-L2 knockdown inhibited osteosarcoma pulmonary metastasis by the RhoA-ROCK-LIMK2 pathway (11); thus, we further investigated the effect of apatinib on the RhoA-ROCK-LIMK2 pathway. As Figure 4A shows, apatinib treatment also attenuated the activation of p-cofilin and p-LIMK2 in KHOS and U2OS cells.

Furthermore, we transfected shRNA targeting VEGFR2 into these two cell lines to confirm the effects of apatinib on the expression of PD-L2 and activation of RhoA-ROCK-LIMK2 signaling. As shown in Figure 4B, VEGFR2 knockdown attenuated the expression of PD-L2, p-LIMK2 and p-cofilin in KHOS and U2OS cells.

Previous studies showed that STAT3 could regulate PD-L1/PD-L2 expression (17–20). VEGFR2 inhibition could induce inactivation of STAT3 in this study (Figures 4A,B). Therefore, we transfected siRNA targeting STAT3 into the two cell lines and tested the effects of STAT3 knockdown on the expression of PD-L2 and activation of RhoA-ROCK-LIMK2 signaling. As shown in Figure 4C, STAT3 knockdown decreased the expression of PD-L2, p-LIMK2 and p-cofilin in KHOS and U2OS cells. KHOS cells that were transfected by VEGFR2 overexpression plasmid increased Y-27632 2HCl-induced decline of p-LIMK and p-Cofilin (Figure 4D), indicating that VEGFR2 promoted migration and invasion by activating RhoA-ROCK-LIMK2 pathway. Additionally, bioinformatics analysis indicated that there may be a coexistence phenomenon between STAT3 and RhoA (Figure 4E). A reduction in RhoA activation in the U2OS-siSTAT3 group compared with the controls was observed via the GTPase assay (Figure 4F).

In conclusion, these data strongly suggest that VEGFR2 inhibition suppresses PD-L2 expression and the RhoA-ROCK-LIMK2 pathway by targeting STAT3.

### VEGFR2 and STAT3 Inhibition Affects Cytoskeletal Rearrangement in Osteosarcoma Cells

The actin cytoskeleton is very important in cell motility, and LIMK and cofilin are key regulators in this process. Phosphorylation of cofilin suppresses its ability to adhere to actin filaments, which inhibits filament breakdown. In addition, cofilin phosphorylation is mediated by LIMK (21–23). Since the phosphorylation of cofilin and LIMK2 was influenced by VEGFR2 or STAT3 expression, as mentioned above, we hypothesized that some morphological alterations occurred when VEGFR2 or STAT3 expression was inhibited.

**FIGURE 5:**
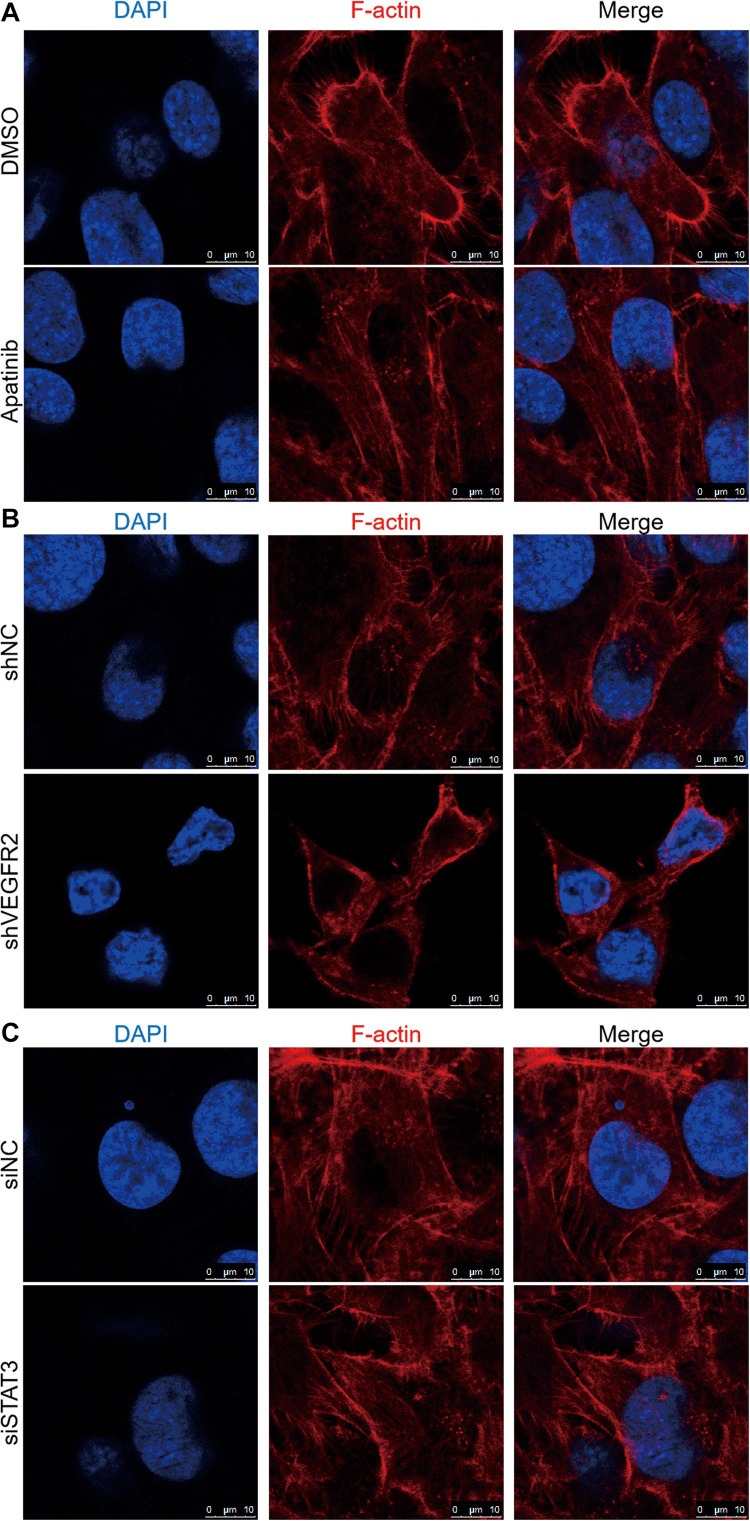
VEGFR2 and STAT3 expression influences osteosarcoma cell cytoskeletal arrangement. The effects of **(A)** apatinib treatment, **(B)** VEGFR2 knockdown and **(C)** STAT3 knockdown on cytoskeletal rearrangement were observed via confocal microscopy in KHOS cells. Representative images are shown. Cell nuclei were stained with DAPI. Scale bar represents 10 μm.

As Figure 5A shows, obvious lamellipodial prominences were observed near the submembranous part in the control group, while the opposite result was observed in the apatinib treatment group, which indicated rearranged cytoskeleton and well-arranged F-actin in cells. In the same manner, compared with those in the KHOS-shVEGFR2 group, the lamellipodial prominences and colored F-actin filaments gathered in the margin of the KHOS-shNC group (Figure 5B). Additionally, F-actin was mostly arranged in KHOS-siSTAT3 cell cytoplasm; however, F-actin and lamellipodial prominences were concentrated near the edge in the KHOS-siNC group (Figure 5C).

These results suggest that the inhibition of VEGFR2 or STAT3 expression has a distinct effect on the cytoskeletal rearrangement of osteosarcoma cells.

### VEGFR2 Inhibition Attenuates Osteosarcoma Cell Metastasis *in vivo*

To confirm the effects of apatinib treatment or VEGFR2 knockdown on the metastatic ability of KHOS cells, corresponding KHOS cell lines were injected intravenously via the tail vein of BALB/c nude mice as described in the tumor xenograft section.

**FIGURE 6:**
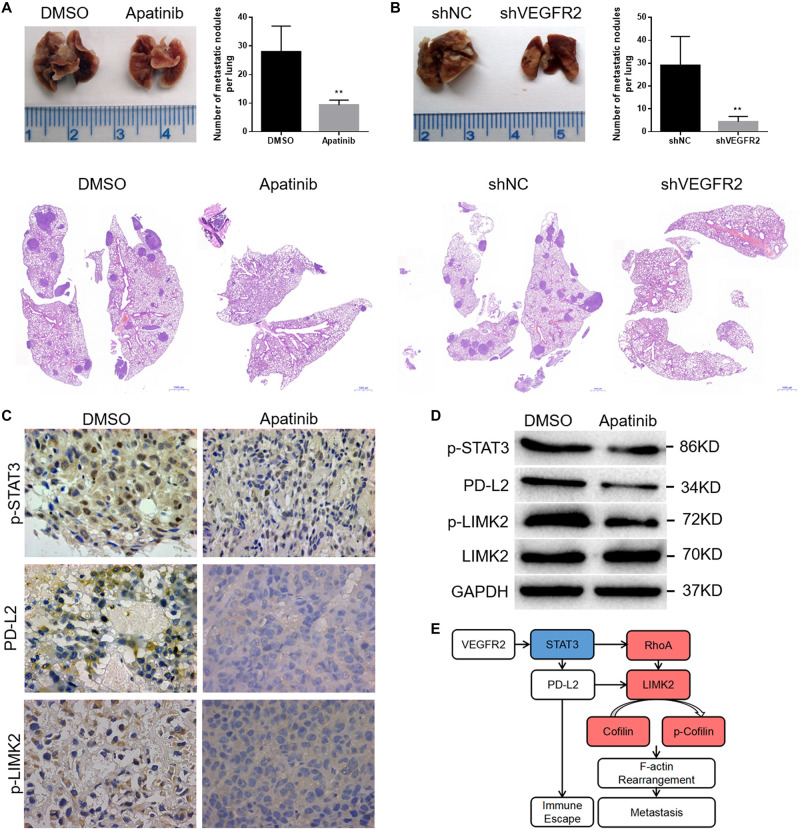
VEGFR2 inhibition attenuates osteosarcoma cell metastasis *in vivo*. **(A,B)** The quantities of pulmonary metastases in the apatinib and DMSO groups or shNC and shVEGFR2 groups are presented. Representative images are shown. **(C,D)** Local primary osteosarcoma was further estimated by immunoblotting and IHC (magnification at 400×) for PD-L2, p-STAT3 and p-LIMK expression. **(E)** A schematic drawing of VEGFR2 in osteosarcoma metastasis. (mean ± S.D., *n* = 4) **p < 0.01.

The apatinib treatment group showed distinctly fewer pulmonary metastases than the control group (Figure 6A). Similarly, lung metastases were more often observed in the control group than in the shVEGFR2 group (Figure 6B). Representative H&E images are shown (Figures 6A,B).

We previously found that there was no significant difference in the tumor size of primary osteosarcoma between the apatinib and DMSO groups (12), and here the primary tumors were further assessed via IHC and WB assays. Consistent with the above results, decreased p-STAT3, PD-L2 and p-LIMK2 expression was observed in the apatinib group compared with the DMSO group (Figures 6C,D).

In conclusion, VEGFR2 inhibition attenuates human osteosarcoma cell metastasis.

## Discussion

Osteosarcoma is a local invasive malignancy, and the incidence of pulmonary metastasis is high. Metastasis is the dominant element influencing the treatment effect in osteosarcoma patients, which is a very complex course involving various molecular biological mechanisms.

In this study, we demonstrate that VEGFR2 expression is higher in pulmonary metastases than in primary specimens using IHC. We also investigated PD-L2 expression in these paired osteosarcoma specimens, and the results indicated that PD-L2 expression is also enhanced in pulmonary metastasis compared to the primary specimen. Moreover, a significant positive relationship between PD-L2 and VEGFR2 expression was confirmed. In addition to its external immune escape effects of tumor cells, PD-L2 also has a few internal effects in tumor cell processes, including EMT, metastasis and autophagy (10, 11). These results indicate that both VEGFR2 and PD-L2 may play important roles in the pulmonary metastasis of osteosarcoma cells. In this study, we show that both apatinib treatment and VEGFR2 knockdown inhibit KHOS and U2OS cell migration and invasion. In the future, we will continue investigating their underlying mechanisms.

To determine the potential elements that support VEGFR2 associations with metastasis, the mRNA expression of 10 VEGFR2-relevant genes in osteosarcoma specimens was measured and observed via bioinformatics analysis. Gene expression methods, GSEA and gene annotation network analysis of VEGFR2-relevant genes were performed. For angiopoiesis, the results show that VEGFR2 may be related to the immune response, cytoskeletal rearrangement and relevant metastasis pathways.

To verify the bioinformatics analysis results, we investigated the effect of VEGFR2 inhibition on the signaling pathway in osteosarcoma. Apatinib (1.5 μM) and shRNA targeting VEGFR2 were used. Similar to the previous results, both apatinib treatment and VEGFR2 knockdown attenuated PD-L2 expression in these two cell lines, consistent with the IHC results. These data indicate that VEGFR2 actively regulates PD-L2 expression in osteosarcoma.

Previous studies have demonstrated that the cell actin cytoskeleton plays a considerable role in adhesion and motility, and cofilin and LIMK are critical regulators (21–23). A previous study indicated that PD-L2 knockdown inhibits the ability of osteosarcoma cells to metastasize by the RhoA-ROCK-LIMK2 signaling pathway (11); thus, we further investigated the effect of VEGFR2 inhibition on RhoA-ROCK-LIMK2 signaling. As shown in Figure 4B, both apatinib treatment and VEGFR2 knockdown decreased p-cofilin and p-LIMK2 expression in KHOS and U2OS cells.

Previous studies suggest that STAT3 could regulate PD-1 ligand expression (17–20). In previous research, we demonstrated that apatinib inhibits PD-L1 presentation by targeting STAT3 in osteosarcoma. Recently, with the promising curative effect of ICIs, it has been proposed that ICI therapy together with antiangiogenic therapy may show a collaborative impact (16). Nevertheless, the potential mechanism remains unclear. In this study, we reveal that STAT3 knockdown attenuates the expression of PD-L2, p-LIMK2 and p-cofilin in osteosarcoma, suggesting that VEGFR2 inhibition suppresses migration, invasion and PD-L2 expression by targeting the STAT3 and RhoA-ROCK-LIMK2 pathways.

It is well known that angiogenesis is related to tumor metastasis (24–26), but its relationship with immune escape remains poorly understood. At present, there are few studies investigating the connection between PD-L2 and VEGFR2. In this sense, we take the lead in revealing, through this study, that VEGFR2 inhibition decreases PD-L2 expression in osteosarcoma cells. Thus, through the tumor cell extrinsic and intrinsic functions of PD-L2, VEGFR2 can affect both osteosarcoma cell metastasis and immune escape through the deactivation of STAT3 and the RhoA-ROCK-LIMK2 pathway (Figure 6E).

Overall, the pro-metastatic functional mechanism of VEGFR2 in osteosarcoma is presented herein. VEGFR2 inhibition targeted the RhoA-ROCK-LIMK2 pathway and STAT3 both *in vitro* and *in vivo*, through which migration, invasion and PD-L2 expression were reduced. It is important to verify the efficacy and mechanism of antiangiogenesis therapy combined with immunotherapy in a humanized mouse model, which may be one of the shortcomings of our study, and we will verify the above results in our further study. Based on the above, our understanding of the regulatory characteristics of angiogenesis in the immune escape of osteosarcoma cells was improved, which offers a feasible strategy for treating metastatic osteosarcoma.

## Data Availability Statement

Publicly available datasets were analyzed in this study, these can be found in the NCBI Gene Expression Omnibus (http://www.ncbi.nlm.nih.gov/geo) (GSE21257 and GSE33382).

## Ethics Statement

The studies involving human participants were reviewed and approved by the Ethics Committees of The Affiliated Hospital of Qingdao University and Peking University People’s Hospital. Written informed consent to participate in this study was provided by the participants’ legal guardian/next of kin. The animal study was reviewed and approved by the Ethics Committees of The Affiliated Hospital of Qingdao University and Peking University People’s Hospital.

## Author Contributions

BZ, BY, and WG planned and designed the study. BZ and CZ were carried out the relevant experiments. BZ and GQ analyzed the data. CR and PY were material contributors. BZ prepared the manuscript. All authors unanimously approved the final draft.

## Conflict of Interest

The authors declare that the research was conducted in the absence of any commercial or financial relationships that could be construed as a potential conflict of interest.

## Abbreviations

ICI: immune checkpoint inhibitor
LIMK2: LIM domain kinase 2
PD-1: programmed death-1
PD-L1: programmed death ligand-1
PD-L2: programmed death ligand-2
STAT3: signal transducer and activator of transcription 3
VEGFR2: vascular endothelial growth factor receptor-2.
